# Supplementary material for: Giant gate-controlled odd-parity magnetoresistance in one-dimensional channels with a magnetic proximity effect
Source: Nat Commun. 2022 Nov 9;13:6538. doi: 10.1038/s41467-022-34177-w (PMC9646711; doi:10.1038/s41467-022-34177-w)
Supplement: Supplementary file 1 — Supplementary Information [file 41467_2022_34177_MOESM1_ESM.pdf]

Supplementary Information

Giant gate-controlled odd-parity magnetoresistance in one-dimensional channels with a magnetic proximity effect

Kosuke Takiguchi[1], Le Duc Anh[1,2,3,*], Takahiro Chiba[4], Harunori Shiratani[1], Ryota Fukuzawa[1,5], Takuji Takahashi[5,6] and Masaaki Tanaka[1,6,7,*]

[1] *Department of Electrical Engineering and Information Systems, The University of Tokyo, Bunkyo-ku, Tokyo 113-8656, Japan.*
[2] *Institute of Engineering Innovation, The University of Tokyo, Bunkyo-ku, Tokyo 113-8656, Japan.*
[3] *PRESTO, Japan Science and Technology Agency, Kawaguchi, Saitama, 332-0012, Japan*
[4] *National Institute of Technology, Fukushima College,Iwaki, Fukushima, 970-8034, Japan*
[5] *Institute of Industrial Science, The University of Tokyo, Meguro-ku, Tokyo 153-8505, Japan*
[6] *Institute for Nano Quantum Information Electronics, The University of Tokyo, Meguro-ku, Tokyo 153-8505, Japan*
[7] *Centre for Spintronics Research Network, The University of Tokyo, Bunkyo-ku, Tokyo 113-8656, Japan.*

Supplementary Note 1: Oscillating behaviour of the OMR

As shown in Fig. 2b, the odd-parity magnetoresistance (OMR) curves show oscillating behaviour. This phenomenon can be explained by Landau quantization. In our theoretical model, Boltzmann's equation describes the OMR in a 1D case as follows.

$$\sigma_{xx} \simeq \frac{e^2}{h}\tau_+|\lambda_{\text{side}}|\sqrt{1+\frac{2E_F}{m^*\lambda_{\text{side}}^2}}\left[1+\alpha-(1-\alpha)\frac{|\lambda_{\text{side}}|}{\lambda_{\text{side}}}\frac{g\mu_B}{2E_F+m^*\lambda_{\text{side}}^2}B_z\right] \quad (4)$$

In this equation, the electrical conductivity σ_{xx} is proportional to the relaxation time of τ_+, where the subscript "+" indicates that τ_+ is the relaxation time of the higher band E_+ (see Fig. 4a).

Generally, the external magnetic field quantizes the density of states (DOS) (Landau quantization), leading to the quantum (SdH) oscillation in the $\sigma_{xx} - B$ characteristics. Since the relaxation time τ is proportional to DOS, τ can be described as

$$\frac{1}{\tau(E,B)} = \frac{1}{\tau_0}\left(1+\frac{\Delta D}{D_0}\right) \quad (S1)$$

where τ_0 represents the relaxation time that is independent of the electric field E and magnetic field B, and $\Delta D/D_0$ represents the B-dependent oscillation component of DOS. Since τ_+ is obtained from eq. (S1), via this relaxation time $\tau(E,B)$, the Landau quantization can manifest itself as oscillation in the 1D transport and the OMR.

According to the Lifshitz-Kosevich theory,[S1,S2] the quantum oscillation becomes clear when the coherence length is long and the mobility is high. This is indeed confirmed in our new sample with higher mobility (= 1.9×10^3 cm^2/Vs) than the previous sample (= 9.4×10^2 cm^2/Vs), as shown in Supplementary Fig. 2. The odd component exhibits much clearer oscillation than the previous sample, which supports our conclusion that the oscillation in OMR originates from the Landau quantization.

Supplementary Note 2: Possible origin of the current dependence of the OMR around I_C = 200 nA

In the InAs channel, there are parallel conductions in the edge (one dimensional (1D)) and center (two-dimensional (2D)) channels (see Supplementary Fig. 5a). In both 1D and 2D channels, there are magnetic proximity effects (MPE) induced by the perpendicular magnetization component M_z of the underlying (Ga,Fe)Sb, as presented in our previous work[S3]. However, we expect that the MPE occurs more strongly in the center 2D channel than in the edge 1D channels. This is because the M_z component is smaller in the edges of (Ga,Fe)Sb where the magnetic moment of Fe usually is tilted towards the side surface. Therefore, we think that *the step-like increase of OMR at I_C = 200 nA (Fig. 2a) is possibly caused by the sudden enhancement of the MPE in the edge due to expansion of the electron wavefunctions in the 1D edge channel towards the 2D center channel at this critical current value.*

As illustrated in Supplementary Fig. 5b, in the 1D edge channel, electron wavefunctions are confined by a triangular potential at the side surfaces and have limited penetration to the 2D center channel. When we increase I, however, the current is more concentrated in the edge, which has higher conductivity because of weaker magnetic scattering from MPE. This increases the electron carrier concentration in the edge. These changes may eventually lead to the occupation of the next quantized level at a slightly higher energy, whose electron wavefunction overlaps more largely with the 2D channel

due to the weaker confinement. This enhances the 1D (edge) - 2D (center) wavefunction overlapping and consequently increases the MPE in the edge channel in a sudden manner as observed at I_C = 200 nA, leading to the sudden increase in $\Delta R/R_0$, as shown in Fig. 2a. With more magnetic scattering in the edge transport, this also explains the slight increase of the total resistance R_0 at I_C in Fig. 2a.

Supplementary Note 3: Hall-bar-orientation dependence of OMR

The Hall-bar orientation (current direction) presented in this paper is always along the $[\bar{1}10]$ axis of the GaAs substrate. In order to investigate the effect of crystal orientation, we fabricate a Hall bar device aligned along the [110] direction and compare its magnetotransport data with those of the Hall bar device aligned along the $[\bar{1}10]$ direction (The device aligned along $[\bar{1}10]$ is the same as D_L). Supplementary Fig. 6a shows temperature dependence of the four-terminal resistance R_{23} in the two devices where the current I is applied parallel to [110] and $[\bar{1}10]$, respectively. The resistance at each T differs between the two Hall bars with different orientations. Also, the magnetoresistance measurements exhibit different OMR magnitude as shown in Supplementary Fig. 6b. The OMR magnitude (= $[R_{23}(1\ \mathrm{T}) - R_{23}(-1\ \mathrm{T})]/2R_{23}(0\ \mathrm{T})$) of [110] and $[\bar{1}10]$ are 0.065% and 0.036%, respectively.

According to the previous study of the Rashba and Dresselhaus effects of InAs/GaSb[S4], the Dresselhaus effect is relatively small, less than one-sixth of the Rashba effect. Thus, it is unlikely that the differences in R_{23} and the OMR magnitude originate from the Dresselhaus effect. One possible reason is the anisotropic distribution of the Fe atoms in the (Ga,Fe)Sb layer with an Fe content of ~20% along the two directions, [110] and $[\bar{1}10]$. In heavily Fe-doped (Ga,Fe)Sb (Fe > ~20%) such as that in our InAs/(Ga,Fe)Sb samples, it is known that spinodal decomposition occurs, leading to fluctuation in the local Fe concentration in the host GaSb crystal[S5]. This Fe-rich (Ga,Fe)Sb regions can favorably form in one direction, [110] or $[\bar{1}10]$[S6]. If this is the case, it can lead to different strength of the magnetic proximity effect (MPE) when the electron carriers in InAs flow in different directions, which will lead to anisotropic OMR.

Supplementary Note 4: Equivalent circuit model of two- and four-terminal magnetotransport measurement

The two results shown in Fig. 2b and c can be understood by the equivalent circuit model shown in Supplementary Fig. 7. We describe the 4-terminal resistance as the sum of odd and even components. Here, the 4-terminal resistances facing each other (R_{23} and R_{65}) are given by

$$R_{23}(B) = R_{23}^{\mathrm{odd}}(B) + R_{23}^{\mathrm{even}}(B) \tag{S2}$$

and

$$R_{65}(B) = R_{65}^{\mathrm{odd}}(B) + R_{65}^{\mathrm{even}}(B) \tag{S2′}$$

Here, we assume that the even components are common in these two resistances ($R^{\mathrm{even}}{}_{23}(B) = R^{\mathrm{even}}{}_{65}(B)$). Reflecting the edge transport data shown in Fig. 2b, the odd components $R_{23}{}^{\mathrm{odd}}$ and $R_{65}{}^{\mathrm{odd}}$ satisfy

$$R_{23}^{\mathrm{odd}}(B) = -R_{65}^{\mathrm{odd}}(B) \tag{S3}$$

Also, the 2D conduction does not show the odd component:

$$R_{2D}(B) = R_{2D}(-B) \tag{S4}$$

The 2-terminal resistance R_{14} can be described as

$$\frac{1}{R_{14}(B)} = \frac{1}{R_{23}^{\text{odd}}(B) + R_{23}^{\text{even}}(B)} + \frac{1}{R_{65}^{\text{odd}}(B) + R_{65}^{\text{even}}(B)} + \frac{1}{R_{2D}(B)} \quad \text{(S5)}$$

Using eq. (S4) and (S5),

$$R_{14}(B) = \frac{R_{2D}(B)[(R_{23}^{\text{even}}(B))^2 - (R_{23}^{\text{odd}}(B))^2]}{2R_{23}^{\text{even}}(B)R_{2D}(B) + [(R_{23}^{\text{even}}(B))^2 - (R_{23}^{\text{odd}}(B))^2]} \quad \text{(S6)}$$

Therefore, R_{14} is an even function of B.

Supplementary Note 5: Counterevidence of intermixing from the Hall resistance

Although the four-terminal measurement can avoid the extrinsic resistance in the transport measurement, it may have the possibility of intermixing of the Hall resistance and the longitudinal resistance. To check the Hall effect contribution as a possible origin of the OMR, we conducted two-terminal magnetotransport measurement, and compared current and gate voltage dependence of the Hall effect to those of OMR.

We measured the two-terminal resistance ($R_{23} = V_{23}/I_{23}$) in two InAs/(Ga,Fe)Sb devices as shown in Fig. 3a and b, where the dashed lines denote the outlines of the Hall bars. Device D1 in Fig. 3a is a Hall bar where the Au pads slightly touch on the edges, while device D2 in Fig. 3b is a Hall bar with branches in full contact with the Au pads. In D1, the two-terminal resistance R_{23} contains large contact resistances, thus exhibiting a large parabolic MR as shown in the upper panel of Supplementary Fig. 8a. Nevertheless, an OMR is observed as shown in the bottom panel of Supplementary Fig. 8a. The small OMR is caused by the high contact resistances due to the small contact areas of the Au electrodes. On the other hand, in device D2, as shown in Supplementary Fig. 8b, the OMR becomes dominant even at small magnetic fields of ±1 T, because the contact resistances are much lower. Therefore, these experiments show that the OMR effect appears not only in the four-terminal but also in the two-terminal configurations.

Also, the current dependence of the Hall resistance ($=V_{26}/I_{14}$) indicates that the Hall resistance and OMR have different origins. The Hall resistance measured at different current magnitudes does not change with current as shown in Supplementary Fig. 9a. We note that the jumps in the Hall resistance data near zero magnetic field are artifacts from our lock-in measurements, not intrinsic properties of the sample. Close to zero magnetic field, when the Hall voltage switches from positive (negative) to negative (positive), the small output voltage makes the phase-offset unstable. This causes the jumps in the Hall resistance data near zero magnetic field in the AC lock-in measurements. For comparison, one can see that the Hall resistance data measured using a DC current does not show such jumps, as shown in Fig. Supplementary Fig. 9b. In our discussions, the values of Hall resistance and odd-parity magnetoresistance (OMR) were obtained at high magnetic field (B ~1 T), where the abovementioned artifacts are irrelevant. Within these reliable values at high magnetic field, the Hall resistances are unchanged regardless of the current magnitude. This current dependence is completely different from those of the longitudinal resistance R_0 and the OMR, which clearly show a jump at 200 nA, as shown in Fig. 2a in the main manuscript. Thus, the different current dependence indicates that the Hall resistance and the OMR have different origins.

Furthermore, V_g dependence of the Hall resistance of D2 is also different from that of OMR: As shown in Fig. 3d, the sign change of OMR is seen by the G1 gate voltage application, whereas the Hall resistance shows negative slope in every V_g value (= +7, 0,

−7 V) as shown in Supplementary Fig. 9c. These results strongly indicate that the OMR is *not* originated from the Hall effect.

It is also worth noting that in InAs the Fermi energy pinning position is above the conduction band bottom at its interface[S7,S8]. Therefore, the interface between a metal electrode and an n-type InAs forms an Ohmic contact. Thus, non-Ohmic behavior caused by the Au/InAs contacts in our device is very unlikely; we actually confirmed Ohmic contacts in our devices.

Supplementary Note 6: Comparison of the 1D and 2D transport via the device size effect

To estimate each contribution of the 1D edge and 2D layer transport channels, we performed transport measurements on two Hall bars, D_L and D_S, with different sizes (l_{14}, l_{23}, w) = (600 μm, 200 μm, 100 μm) and (180 μm, 60 μm, 30 μm), respectively. Here w is the width of the Hall bar and l_{14}, l_{23} are the distances between terminals "1" to "4" and "2" to "3", respectively, as shown in Supplementary Fig. 11a. It is highly challenging to control the 1D and 2D conduction independently using the top gate voltage because the width of the 1D channel is too narrow. As described below, we compare the magnetotransport results and OMRs in the two Hall bars without applying a gate voltage.

As shown in Supplementary Fig. 11b, temperature (T) dependence of the four-terminal resistance R_{23} shows significant difference between D_L and D_S. Because the ratios w:l_{23} of D_L and D_S are the same (=1:2), R_{23} should be equal in D_L and D_S if the electrical conduction is uniform. However, the experimental $R_{23} – T$ curves differ between the two devices, which suggests that the electrical transport is non-uniform due to the coexistence of the 1D and 2D channels. As shown in the inset of Supplementary Fig. 11c, the transport results can be understood by a simple resistor network model, where the resistors corresponding to the 2D (R_{2D}) and 1D (R_{1D}) channels are connected in parallel. Assuming that the 1D channel has the same width in D_L and D_S, the total resistance of the network can be expressed as:

$$(R_{23}(0\ \mathrm{T}))^{-1} = \left(r_{2D}\frac{l_{23}}{w}\right)^{-1} + (r_{1D}l_{23})^{-1} \quad \text{(S7)}$$

where $R_{2D} = r_{2D}(l_{23}/w)$, and $R_{1D} = r_{1D}l_{23}$. By solving simultaneous equations for r_{2D} and r_{1D} with D_L and D_S at each temperature, we obtain separate $R_{2D} – T$ and $R_{1D} – T$ curves as shown in Supplementary Fig. 11c. Note that Supplementary Fig. 11c shows the case of D_L. At 3.8 K, the ratio R_{1D}/R_{2D} is 3.2, which corresponds to the current distribution ratio between the 1D and 2D channels. Thus, *2D and 1D transport channels coexist in the InAs/(Ga,Fe)Sb heterostructures, and the 1D transport does not dominate*.

One interesting observation is that the behavior of the $R_{2D} – T$ and $R_{1D} – T$ curves below 10 K agree with our model presented in Supplementary Note 2. In this Note, we argued that there is scattering between in the 1D and 2D channel, where the 2D channel has much stronger MPE than the 1D channel. As shown in Supplementary Fig. 11c, the 2D channel resistance exhibits an increase as temperature decreases below 10 K, which follows the logarithmic trend that is characteristic of the Kondo effect. This suggests strong scattering with magnetic impurities at the InAs/(Ga,Fe)Sb interface in the 2D channel. In contrast, the 1D channel resistance decreases as temperature decreases, exhibiting metallic conduction. This fact implies that the electrons in the 2D channel feel

stronger MPE from the localized spins in the (Ga,Fe)Sb layer underneath, just as we expected.

The magnetotransport data of R_{23} (four-terminal resistance) are shown in the Supplementary Fig. 11d. The OMR magnitudes at 1 T (=[R_{23}(1 T) − R_{23}(−1 T)]/2R_{23}(0 T)) of D_L and D_S are 0.037% and 0.095%, respectively. The OMR decreases with increasing the Hall bar width w, which is reasonable considering our scenario of parallel conduction between the 1D and 2D channels: When w increases, the conduction of the 2D channel, which does not show OMR, becomes more dominant. Thus, the ratio between the resistance change due to OMR in the 1D edge channel versus the total resistance becomes smaller, leading to a smaller OMR.

This can be proved analytically as described below. Using eq. (S7), the OMR magnitude is expressed as

$$\frac{R_{23}(1\,\mathrm{T})-R_{23}(-1\,\mathrm{T})}{2R_{23}(0\,\mathrm{T})}$$

$$=\frac{\left[\left(R_{2\mathrm{D}}(1\,\mathrm{T})\right)^{-1}+\left(R_{1\mathrm{D}}(1\,\mathrm{T})\right)^{-1}\right]^{-1}-\left[\left(R_{2\mathrm{D}}(-1\,\mathrm{T})\right)^{-1}+\left(R_{1\mathrm{D}}(-1\,\mathrm{T})\right)^{-1}\right]^{-1}}{2\left[\left(R_{2\mathrm{D}}(0\,\mathrm{T})\right)^{-1}+\left(R_{1\mathrm{D}}(0\,\mathrm{T})\right)^{-1}\right]^{-1}}$$

$$=\frac{\left[\left(wr_{2\mathrm{D}}(1\,\mathrm{T})\right)^{-1}+\left(r_{1\mathrm{D}}(1\,\mathrm{T})\right)^{-1}\right]^{-1}-\left[\left(wr_{2\mathrm{D}}(-1\,\mathrm{T})\right)^{-1}+\left(r_{1\mathrm{D}}(-1\,\mathrm{T})\right)^{-1}\right]^{-1}}{2\left[\left(wr_{2\mathrm{D}}(0\,\mathrm{T})\right)^{-1}+\left(r_{1\mathrm{D}}(0\,\mathrm{T})\right)^{-1}\right]^{-1}} \quad \text{(S8)}$$

The differential of eq. (S8) with w is

$$\frac{\partial}{\partial w}\left(\frac{R_{23}(1\,\mathrm{T})-R_{23}(-1\,\mathrm{T})}{2R_{23}(0\,\mathrm{T})}\right)$$

$$=\frac{\partial}{\partial w}\left(\frac{[(h'w)^{-1}+g_+^{-1}]^{-1}-[(h'w)^{-1}+g_-^{-1}]^{-1}}{2[(h_0w)^{-1}+g_0^{-1}]^{-1}}\right)$$

$$=-\frac{(g_--g_+)\left(h_0(h'^2w^2-g_+g_-)+h'g_0(2h'w+g_++g_-)\right)}{2(h'w+g_+)^2(h'w+g_-)^2} \quad \text{(S9)}$$

where $1/r_{1\mathrm{D}}(\pm 1\,\mathrm{T})=g_\pm$, $1/r_{1\mathrm{D}}(0\,\mathrm{T})=g_0$, $1/r_{2\mathrm{D}}(\pm 1\,\mathrm{T})=h'$, and $1/r_{2\mathrm{D}}(0\,\mathrm{T})=h_0$.

Note that since the 2D channel does not show the OMR component, $R_{2\mathrm{D}}(1\,\mathrm{T})=R_{2\mathrm{D}}(-1\,\mathrm{T})$, *i.e.* $r_{2\mathrm{D}}(1\,\mathrm{T})=r_{2\mathrm{D}}(-1\,\mathrm{T})=1/h'$.

Here we prove that eq. (S9) is always negative at $w > 0$. For the first bracket on the numerator, since the OMR magnitude in eq. (S8) is defined as positive, $R_{1\mathrm{D}}(1\,\mathrm{T}) > R_{1\mathrm{D}}(-1\,\mathrm{T})$, *i.e.* $r_{1\mathrm{D}}(1\,\mathrm{T}) > r_{1\mathrm{D}}(-1\,\mathrm{T})$. Therefore, $g_- - g_+ > 0$. Also, for the second bracket on the numerator, since all the parameters are positive, it is sufficient to prove $h'^2w^2 - g_-g_+ > 0$. While $R_{1\mathrm{D}}(0\,\mathrm{T})/R_{2\mathrm{D}}(0\,\mathrm{T}) = 3.2$, the MR magnitude at ±1 T is less than 6% as shown in Supplementary Fig. 11d, which implies $R_{2\mathrm{D}}$(±1 T) < $R_{1\mathrm{D}}$(±1 T). Thus,

$$R_{2\mathrm{D}}(1\,\mathrm{T})R_{2\mathrm{D}}(-1\,\mathrm{T}) < R_{1\mathrm{D}}(1\,\mathrm{T})R_{1\mathrm{D}}(-1\,\mathrm{T})$$

$$\frac{\left(r_{2D}(1\,\mathrm{T})\right)^2}{w^2} < r_{1D}(1\,\mathrm{T})r_{1D}(-1\,\mathrm{T})$$

$$h'^2w^2 - g_-g_+ > 0 \quad \text{(S10)}$$

From the argument described above, the OMR magnitude decreases with increasing *w*. Thus, our model can explain the device size dependence of OMR, indicating that the 1D channel is the main origin of the OMR.

Supplementary Note 7: Theoretical analysis using Boltzmann's equation

The low-energy 1D electrons with a Rashba-type SOI at the edge of a 2D electron gas on a ferromagnetic insulator (FI) are described by the effective Hamiltonian given by eq. (1) in the main manuscript,

$$\widehat{H}_{1\mathrm{D}}(k_x) = \frac{\hbar^2 k_x^2}{2m^*}\sigma_0 + (\Lambda_{\mathrm{side}} k_x + \Delta_z)\sigma_z + \Lambda_{\mathrm{top}} k_x \sigma_y \tag{1}$$

The 2D electrons near the interface are coupled to the magnetic dopants (Fe) in (Ga,Fe)Sb via the *s-d* exchange interaction, described by

$$\widehat{H}_{sd}(\mathbf{r}) = -V_{sd}\sum_i \mathbf{S}_i \cdot \boldsymbol{\sigma}\delta(\mathbf{r} - \mathbf{R}_i) \tag{S11}$$

where V_{sd} is the *s-d* exchange potential, $\mathbf{S}_i$ is the local spin operator, and $\mathbf{R}_i$ is the position operator of the *i*th Fe magnetic dopant. Equation (1) gives the energy dispersion shown in eq. (2),

$$E_s = \frac{\hbar^2 k_x^2}{2m^*} + s\sqrt{(\Lambda_{\mathrm{side}} k_x + \Delta_z)^2 + \left(\Lambda_{\mathrm{top}} k_x\right)^2} \tag{2}$$

where $s = +/-$ denotes the upper and lower bands (see Fig. 4a in the main text), as well as the related eigenstates $\varphi_{ks}(x) = e^{ikx}|u_{ks}\rangle$, with

$$|u_{k+}\rangle = \begin{pmatrix} -i\sin\frac{\theta_k}{2} \\ \cos\frac{\theta_k}{2} \end{pmatrix}, |u_{k-}\rangle = \begin{pmatrix} -i\cos\frac{\theta_k}{2} \\ \sin\frac{\theta_k}{2} \end{pmatrix} \tag{S12}$$

Here, the angle θ_k is defined by

$$\cos\theta_k = \frac{\Lambda_{\mathrm{side}} k_x + \Delta_z}{\sqrt{(\Lambda_{\mathrm{side}} k_x + \Delta_z)^2 + \left(\Lambda_{\mathrm{top}} k_x\right)^2}}, \sin\theta_k = \frac{\Lambda_{\mathrm{top}} k_x}{\sqrt{(\Lambda_{\mathrm{side}} k_x + \Delta_z)^2 + \left(\Lambda_{\mathrm{top}} k_x\right)^2}} \tag{S13}$$

The band diagrams are schematically represented in Fig. 4a, in which the position of the Fermi energy determines the topology of the Fermi surfaces. In the presence of an out-of-plane magnetic field (B_z), the Rashba-type spin splitting becomes asymmetric because of the Zeeman splitting Δ_z. As we mentioned in the main manuscript, due to Λ_{top}, the eigenstate of eq. (2) can be labelled by chirality, which is indicated by green and pink colors in Fig. 4a and b.

Let us calculate the charge current arising from the edge transport. According to eq. (1), the velocity operator is given by

$$\hat{v} = \frac{1}{\hbar}\frac{\partial \widehat{H}_{1D}}{\partial k_x} = \frac{\hbar k_x}{m^*}\sigma_0 - \frac{\Lambda_{\mathrm{side}}}{\hbar}\sigma_z + \frac{\Lambda_{\mathrm{top}}}{\hbar}\sigma_y \tag{S14}$$

The expectation value of Eq. (S14) on each eigenstate corresponds to the electron group velocity

$$v_s^{(0)} = \frac{1}{\hbar}\frac{\partial E_s}{\partial k_x} = \langle u_{ks}|\hat{v}|u_{ks}\rangle = \frac{\hbar k_x}{m^*} + s\frac{\Lambda_{\mathrm{side}}}{\hbar}\cos\theta_k + s\frac{\Lambda_{\mathrm{top}}}{\hbar}\sin\theta_k \tag{S15}$$

where the first term is the normal velocity and the second and third terms are additional velocities induced by the Rashba SOI. Hereafter, we assume Λ_{top} ($=\hbar\lambda_{\mathrm{top}}$) $\ll$ Λ_{side} ($=\hbar\lambda_{\mathrm{side}}$), which means that the electric field at the side edges is much larger than that at

the top surface, and neglect the effect of the Rashba SOI from the interface on the energy dispersion.[S9]

Let us now calculate the charge current driven by an electric field E_x. When the electric field is applied, under the relaxation time approximation, the Fermi surface shifts by $\delta k_x = -eE_x\tau_s/\hbar$, where τ_s is the electron relaxation time. τ_+ and τ_- can differ due to the chirality dependent scattering. (See Supplementary Note 8.)

By taking a power series expansion with respect to the electric field E_x up to the 1st order, the corresponding deviation from the equilibrium distribution function $f_s^{(0)}$ is given by

$$f_s = f_s^{(0)} + f_s^{(1)}(E_x)\,, \tag{S16}$$

where $f_s^{(1)}$ is the first-order deviation from $f_s^{(0)}$. Therefore, the charge current density J_x consists of $J_x^{(0)}$ and $J_x^{(1)}$; $J_x = J_x^{(0)} + J_x^{(1)}(E_x)$. Here, the first-order current density is given by

$$\begin{aligned} J_x^{(1)} &= \sum_s \int \frac{dk_x}{2\pi}\left(-ev_s^{(0)}\right) f_s^{(1)}(E_x) \\ &= -\frac{e^2E_x}{2\pi\hbar}\sum_s \tau_s \int dk_x v_s^{(0)} \frac{\partial f_s^{(0)}}{\partial k_x} \\ &= C_1 \sum_s \tau_s \int dE_s v_s^{(0)}(E_s)\left(-\frac{\partial f_s^{(0)}}{\partial E_s}\right) \end{aligned} \tag{S17}$$

where $C_1 = e^2E_x/2\pi\hbar$. For $T \to 0$, $-\partial f_s^{(0)}/\partial E_s = \delta(E_s - E_F)$; then,

$$\begin{aligned} J_x &= C_1 \sum_s \tau_s v_s^{(0)}(E_F) \\ &= C_1 \sum_s \tau_s |\lambda_E| \left(1 + \frac{2E_F}{m^*\lambda_{\rm side}^2} - s\frac{|\lambda_{\rm side}|}{\lambda_{\rm side}}\frac{2\Delta_z}{m^*\lambda_{\rm side}^2}\right)^{\frac{1}{2}} \\ &= C_1\tau_+|\lambda_{\rm side}|\left[\left(1 + \frac{2E_F}{m^*\lambda_{\rm side}^2} - \frac{|\lambda_{\rm side}|}{\lambda_{\rm side}}\frac{2\Delta_z}{m^*\lambda_{\rm side}^2}\right)^{\frac{1}{2}} + \alpha\left(1 + \frac{2E_F}{m^*\lambda_{\rm side}^2} + \frac{|\lambda_{\rm side}|}{\lambda_{\rm side}}\frac{2\Delta_z}{m^*\lambda_{\rm side}^2}\right)^{\frac{1}{2}}\right] \end{aligned} \tag{S18}$$

For $E_F \gg \Delta_z$, eq. (S18) is approximately rewritten as

$$J_x \simeq C_1\tau_+|\lambda_{\rm side}|\sqrt{1 + \frac{2E_F}{m^*\lambda_{\rm side}^2}}\left[1 + \alpha - (1-\alpha)\frac{|\lambda_{\rm side}|}{\lambda_{\rm side}}\frac{\Delta_z}{2E_F + m^*\lambda_{\rm side}^2}\right] \tag{S19}$$

where

$$\alpha = \frac{\tau_-}{\tau_+} \tag{S20}$$

is a parameter, which results from an asymmetric scattering between the E_- and E_+ bands. The detailed discussion on this parameter is given in Supplementary Note 8. Therefore, the conductivity is given by eq. (4),

$$\sigma_{xx} \simeq \frac{e^2}{h}\tau_+|\lambda_{\rm side}|\sqrt{1 + \frac{2E_F}{m^*\lambda_{\rm side}^2}}\left[1 + \alpha - (1-\alpha)\frac{|\lambda_{\rm side}|}{\lambda_{\rm side}}\frac{g\mu_B}{2E_F + m^*\lambda_{\rm side}^2}B_z\right] \tag{4}$$

where h is the Planck's constant.

Supplementary Note 8: Origin of the asymmetric scattering

While the influence of ferromagnetism on the OMR is common in all the previous reports and ours, there is one fundamental difference between the OMR observed in our InAs/(Ga,Fe)Sb bilayer heterostructure and the others. As shown in Supplementary Table 1, in the previous works, the linear OMR only occurred when the magnetic field **B** and the magnetization **M** are *separately* changed (thus they are not always parallel; $\mathbf{B} \nparallel \mathbf{M}$). This is because, although each of **B** and **M** can break the TRS, simultaneous reversal of both **B** and **M** preserves the TRS. Therefore, when **B** and **M** are completely aligned (**B** // **M**, which is the case when **B** is large), the Onsager's reciprocal theorem requires $\sigma_{xx}(\mathbf{B},\mathbf{M}) = \sigma_{xx}(-\mathbf{B},-\mathbf{M})$ and no OMR is allowed (σ_{xx} is longitudinal conductivity), as discussed in Ref. S16. However, when $\mathbf{B} \nparallel \mathbf{M}$, the TRS is broken by reversing **B** or **M** alone, which relaxes the Onsager's theorem requirement and allows OMR to occur. For example, the OMR in $SmCo_5$[S18.S19] is observed only when **B** is smaller than the coercivity of **M** (~ 2 T). Thus, previously reported OMR phenomena were only realized in small magnetic field regions.

In contrast, the OMR in our InAs/(Ga,Fe)Sb system is fundamentally different. In our case, the OMR is present and linearly proportional to the magnetic field **B** in the whole range of **B** up to 10 T, which is much larger than the coercivity of (Ga,Fe)Sb (~ 50 mT). It is obvious that the magnetization **M** of (Ga,Fe)Sb should closely follow **B** in most of the magnetic field range (*i.e.* **B** // **M**). Therefore, the observation of OMR in our case is striking, because the TRS is preserved when both **B** and **M** are simultaneously reversed and always parallel, as mentioned above. Thus, this suggests that the OMR in our InAs/(Ga,Fe)Sb system should have a different origin and cannot be explained by the same theoretical framework of the previous reports.

At the present stage, we do not have a rigorous theoretical explanation for the large OMR observed in our InAs/(Ga,Fe)Sb system. However, our idea is that there should be some other factors that break the TRS, which only weakly depends on the external magnetic field **B**. Here we show a possible mechanism to explain our OMR results. Figure 4a in the main manuscript illustrates the band dispersions of the 1D edge channel of InAs, where there are two branches of energy dispersion E_+ and E_-. These dispersions are the results of the Rashba spin orbit interaction (SOI) at the top (*z* direction) and side (*y* direction) surface of the InAs edge (see Fig.4a in the main manuscript). The eigenvalues of these E_+ and E_- branches were obtained from the Hamiltonian in eq. (1) and have been given in the main manuscript.

$$\hat{H}_{1\mathrm{D}}(k_x) = \frac{\hbar^2 k_x^2}{2m^*}\sigma_0 + (\Lambda_{\mathrm{side}} k_x + \Delta_z)\sigma_z + \Lambda_{\mathrm{top}} k_x \sigma_y \quad (1)$$

It is important to realize that the spin components σ_y and σ_z of the electron carriers are locked to the momentum k_x in opposite directions between E_+ and E_-. Thus, the + and – subscripts also correspond to the different "chirality" of these bands. In our theoretical model based on the Boltzmann formalism, we proposed a phenomenological parameter, α (= τ_-/τ_+, where τ_+ and τ_- are the relaxation time of electron carriers in the E_+ and E_- states, respectively). If there is asymmetry between τ_+ and τ_- (that is, $\alpha \neq 1$), the OMR is expressed as:

$$\sigma_{xx} \simeq \frac{e^2}{h}\,\tau_+|\lambda_{\mathrm{side}}|\sqrt{1+\frac{2E_F}{m^*\lambda_{\mathrm{side}}^2}}\left[1+\alpha-(1-\alpha)\frac{|\lambda_{\mathrm{side}}|}{\lambda_{\mathrm{side}}}\frac{g\mu_B}{2E_F+m^*\lambda_{\mathrm{side}}^2}B_z\right] \quad (4)$$

This equation can quantitatively reproduce the linear OMR results observed in our experiment when $\alpha \neq 1$, as shown in Fig. 4c and d in the main manuscript. One possible origin of the asymmetric relaxation time between E_+ and E_- can be deduced if we consider the scattering from the 1D edge channel to the 2D channel in the InAs layer, as shown in Supplementary Fig. 5a. In the 2D channel, the spin component σ_y of the electron carriers is also locked to k_x due to the Rashba effect due to the electric field in the z direction. Here we consider that only the scattering within the same chirality is allowed (Supplementary Fig. 15a). The relaxation time of each σ_y direction (τ_+, τ_-) should be different between the + and – chirality due to their different density of states at the Fermi level. Because the chirality + and – are only determined by the Rashba SOI, the definition and magnitude of α do not change even when reversing the z component of **B** (= B_z). This leads to the appearance of OMR even when **B**//**M** (Supplementary Fig. 15b). We note that the σ_z component of electron carriers may not be conserved in the scattering between the 1D and 2D channels because spin-flip scattering events may occur with the existence of localized spins in (Ga,Fe)Sb, which are aligned in the z direction. The current-independence of the OMR in our system can also be explained using the same framework: When reversing the current direction, σ_y in both 1D and 2D channels are flipped, and the definition of α does not change (Supplementary Fig. 15c). Thus, the TRS is broken when focusing only on the 1D channel while the non-reciprocity does not occur when considering both the 1D and 2D channels.

The magnetic proximity effect (MPE) also plays an important role in the scattering process. As we mention in Supplementary Note 2 and 6, the MPE mainly affects the 2D channel by opening a gap (= Δ_{2D}) between different chirality bands as shown in Supplementary Fig. 15a and d; as the MPE is increased, Δ_{2D} is increased. As a result, the energy dispersion is altered by the MPE, which will lead to larger imbalance between τ_+ and τ_- and larger OMR (Supplementary Fig. 15d).

Supplementary Note 9: Correspondence between our theoretical model and experimental results

There are two unique aspects of our OMR, which make it stand out of all the previously reported **B**-odd magnetoresistances (see Supplementary Table 1):

1. Our OMR does not depend on the current direction. This indicates that it is not a non-reciprocal transport phenomenon, which was observed in the non-linear response region[S10,S11,S12, S13].
2. Our OMR appears even when the magnetic field **B** is parallel to the magnetization **M** (**B**//**M**). This is completely different from other linear MR phenomena[S14,S15,S16,S17](observed in the linear response region), where **B** and **M** must be non-collinear.

Thus, the theoretical models proposed thus far to describe the **B**-odd components of magnetoresistance such as the unidirectional MR (observed in the non-linear response region)[S10,S11,S12,S13] and linear MR [S14,S15,S16,S17] (observed in the linear response region) cannot explain the new OMR observed in our study. Therefore, a new theoretical model is required.

Furthermore, there are two important points deduced directly from our experimental results and careful considerations on the mechanism of our OMR:

- First, *the coupling between the edge-center channels plays a vital role in inducing the new OMR,* which is deduced from the following experimental facts:

- The OMR occurs at the edge transport of the InAs layer, which is evident from its absence in the two-terminal measurement and its opposite signs when measured along the opposite edges, as shown in Fig. 2 and discussed in the main manuscript.
- The OMR requires a magnetic proximity effect (MPE) from (Ga,Fe)Sb to InAs, as demonstrated by the global gate control experiment shown in Fig. 3a,c of the main manuscript. The magnetic coupling at the (Ga,Fe)Sb/InAs interface, however, occurs mainly in the center region (2D channel) of the InAs layer. This is evident in the absence and appearance of a Kondo-like tail at $T < \sim 10$ K in the temperature dependence of the resistance of the edge (1D) and center (2D) channels, respectively, as shown in Supplementary Note 6.

- Second, *OMR will appear if there is asymmetry in the scattering rate of electron carriers in the two brands E_+ and E_- of the energy-momentum dispersion relationship (see Fig. 4b in the main manuscript).* This was straightforwardly deduced from the 1D Hamiltonian of the edge channel and the Boltzmann's formalism discussed in the Method section. As shown in Fig. R10, it is also important to point out that these two bands (E_+ and E_-) are characterized by a spin-momentum locking effect with opposite chiralities, due to the Rashba spin-orbit coupling in the InAs edge channel.

All these features are straightforwardly deduced from the experimental results and the Hamiltonian of the InAs edge channel, without any unreasonable assumption.

Therefore, in our proposed model, we figure out that there will be asymmetry in the scattering rate of electron carriers in the two brands (E_+ and E_-) if one considers spin-momentum locking effect in both the edge and center channels, which depends only on the current direction. Here, *we made only one assumption that scattering events between the two channels must occur within the same chirality, which is totally reasonable*. This chirality reservation in the edge-center scattering of InAs yields asymmetric scattering rates ($\tau_+ \neq \tau_-$, where $\tau_\pm$ indicates the relaxation time for the scattering between the 1D – 2D channels of the E_+ and E_- bands), as illustrated in Fig. 4. From this assumption, our theoretical model expects that

(i) OMR in InAs/(Ga,Fe)Sb emerges even when **B**//**M**.
(ii) OMR in InAs/(Ga,Fe)Sb is not affected by reversing the current direction.
(iii) OMR in InAs/(Ga,Fe)Sb can be modulated by varying the MPE.

These notable features are confirmed by our experimental results [(i) Fig. 1c in the main manuscript, (ii) Supplementary Fig. 4, (iii) Fig. 3c in the main manuscript. See Supplementary Note 8 for the detailed discussion]. Therefore, at this stage, our model based on the chirality-dependent scattering between the edge (1D) and center (2D) channels is the first theoretical model that can explain all the aspects of this new OMR. By introducing the chirality-dependent scattering into our Boltzmann formalism, our theoretical model successfully reproduces the linear change against **B** and magnitude of OMR (13.5% at 10 T, see Figs. 4c,d,e in the main manuscript). This shows one of the direct links between our experimental results and our theoretical model.

Supplementary Note 10: Magnetic field sweeping effect on OMR

We conducted the measurement of OMR using different systems, a PPMS with a superconducting magnet ($|B| < 14$ T) and a self-designed system with an electromagnet ($|B| < 1$ T). The magnetic field sweeping speed in the former (PPMS) was typically 0.3 T/min, while that in the latter was roughly 0.1 T/min. The OMR data obtained in these two machines are in good agreement as shown in Supplementary Fig. 16. In addition, the OMR exists in our gate-voltage sweeping experiment with a *constant* magnetic field as shown in Fig. 3c in the main manuscript. Thus, the OMR data are not affected by changing the measurement system or the magnetic field sweeping speed.

Supplementary Table 1. Comparison of OMR observed in previous reports and our work. The maximum OMR magnitude $\Delta R/R_0$ (= $[(R(B)-R(-B))/2]/R(0\ \mathrm{T})$) normalized by R_0 (= $R(0\ \mathrm{T})$) were obtained under magnetic field B at temperature T.

material	$\Delta R/R_0$ (%)	B (T)	T (K)	Observable under B // M	Proposed origin	ref.
$SmCo_5$	1.3×10^{-2}	0.015	room temp.	No	non-uniform distribution of the magnetization	S18
$SmCo_5$	4.6×10^{-2}	0.5	300	No	Zeeman splitting/ anomalous Hall effect	S16,S19
$Gd_2Os_2O_7$	5.0×10^{-2}	2	195	No	magnetic domain walls	S19
$Eu_2Ir_2O_7$ (theory)	-	-	-	No	Berry curvature, magnetic moment, and shift vector	S16
$Eu_2Ir_2O_7$ (experiment)	0.44	9	2	No	magnetic texture	S20
Fe_3GeTe_2 /graphite/ Fe_3GeTe_2	1.1	0.01	50	No	interfacial SOI of Fe_3GeTe_2 as a topological nodal line	S21
**InAs/ (Ga,Fe)Sb**	**13.5<br>5**	**10<br>10**	**2<br>300**	**Yes**	**Rashba SOI at the edge of InAs and magnetic proximity effect**	**Our work**

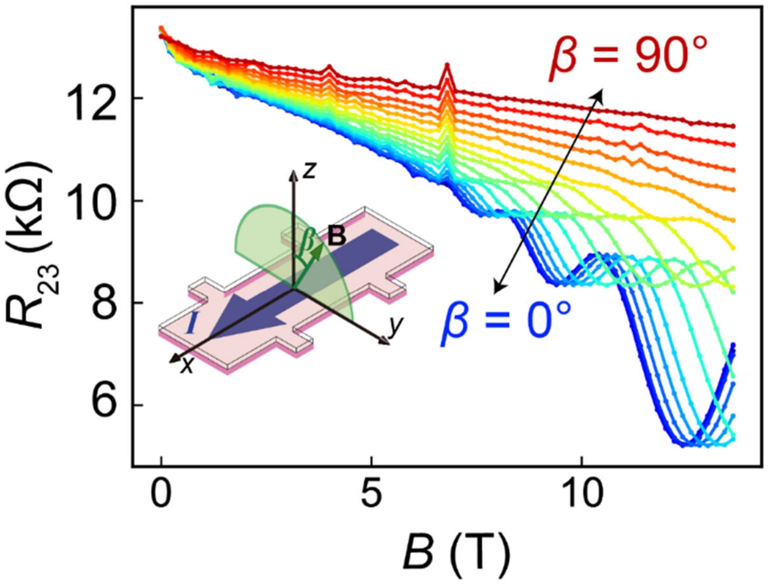

Supplementary Fig. 1| Angular dependence of SdH oscillation. Magnetoresistance of sample A in each β angle from 0° to 90°. The inset shows the definition of β in the *yz* plane.

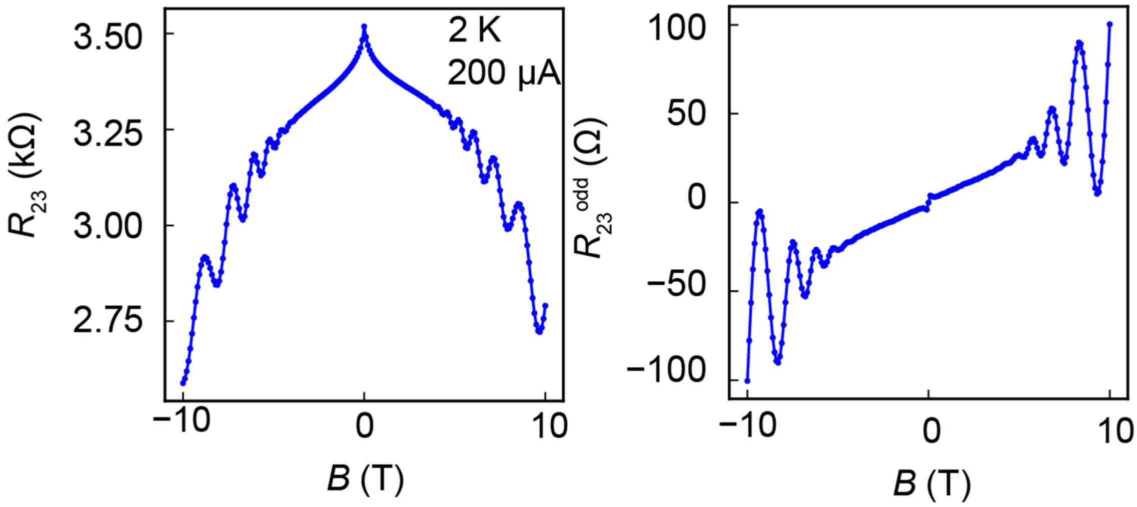

Supplementary Fig. 2| Oscillating components in the OMR of a high mobility sample. Magnetotransport measurement results of a higher mobility sample of the same structure shown in Fig. 1a (left panel) and the odd component (right panel) at 2 K with 200 μA.

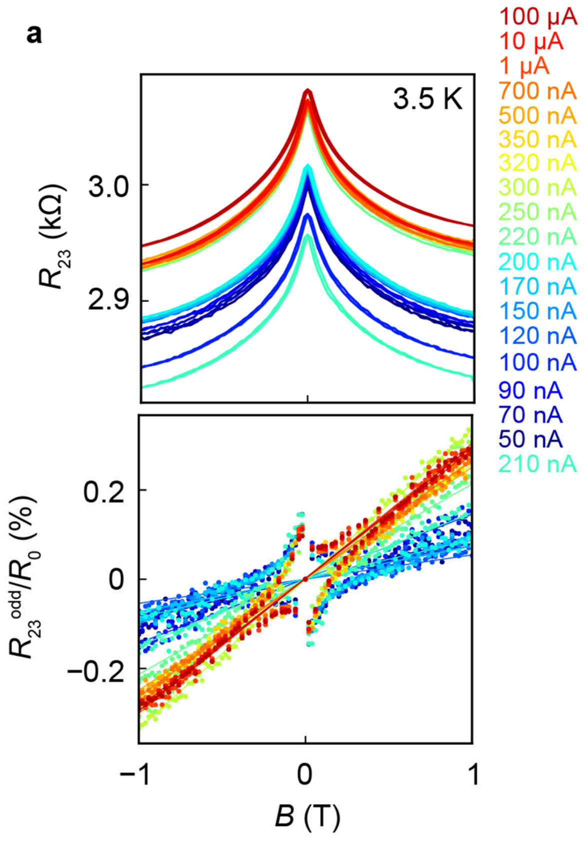

Supplementary Fig. 3| Current dependence of the OMR. **a**, Current dependence of the magnetoresistance curves (upper panel) and the odd components (lower panel) at 3.5 K with perpendicular B. Note that $R_{23}^{\mathrm{odd}}(B)= (R_{23}(B) - R_{23}(-B))/2$. Here, $R_{23}(B)$ is the resistance measured between terminals 2 and 3, and R_0 is $R_{23}(0\ \mathrm{T})$.

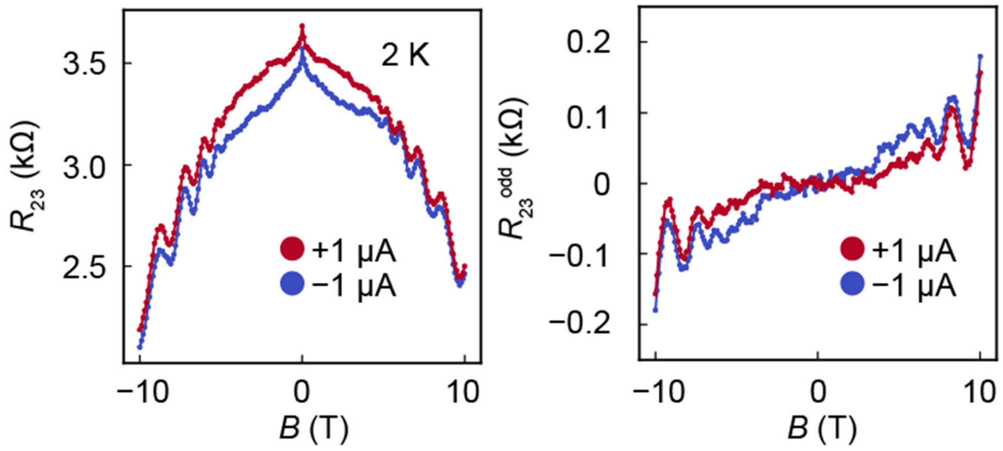

Supplementary Fig. 4| Current direction dependence of the OMR. Magnetoresistance curves with opposite current directions (left panel) and their odd components (right panel) at 2 K with ±1 μA.

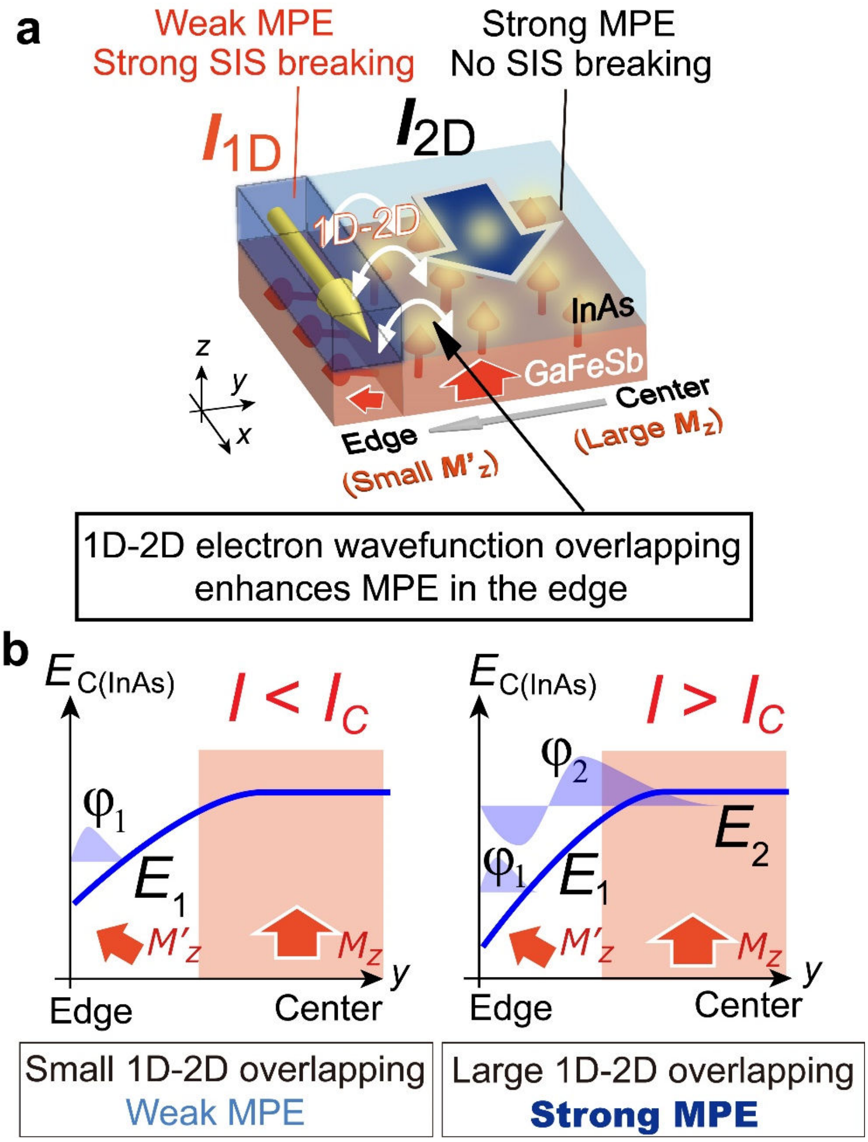

Supplementary Fig. 5| Electronic states and magnetization situation near the side edge a, Schematic illustration of the InAs/(Ga,Fe)Sb bilayer heterostructure near the Hall bar edge. In the (Ga,Fe)Sb layer, the magnetic moment M_z' (red arrows) near the side edge may be canted and does not effectively induce the MPE in the InAs edge channel. On the other hand, in the 2D channel (center) side, the magnetic moment M_z is aligned in the z direction due to the magnetic anisotropy of (Ga,Fe)Sb, leading to strong MPE. Through overlapping of the electron wavefunctions in the 1D channel (edge) with the 2D channel (center), MPE is strongly induced in the 1D channel at $I > I_C$. The MPE, together with the Rashba SOI, leads to the appearance of OMR. **b**, Illustrated electronic subband structure of the conduction band bottom $E_{C(InAs)}$ of InAs near the edge (blue curves). When the current I is increased, the electron carriers are accumulated near the edge. This leads to a change in the occupied quantized levels at I_C. When higher levels are occupied by electrons at $I > I_C$, the 1D wavefunctions largely overlap with the 2D center region, which suddenly enhances the MPE in the edge, leading to the sudden increase of OMR.

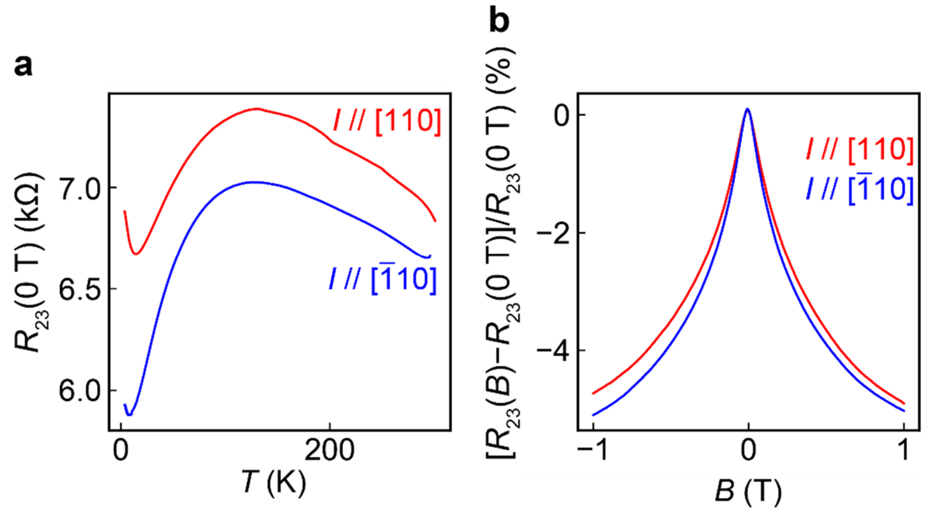

Supplementary Fig. 6| Hall-bar-orientation dependence of electrical transport and OMR. a, Temperature (T) dependence of R_{23}(0T) with I // [110] (red) and I // [$\bar{1}$10] (blue). **b**, Normalized magnetoresistance by the zero-field resistance, [$R_{23}(B)-R_{23}$(0 T)]/ R_{23}(0 T), with I // [110] (red) and I // [$\bar{1}$10] (blue).

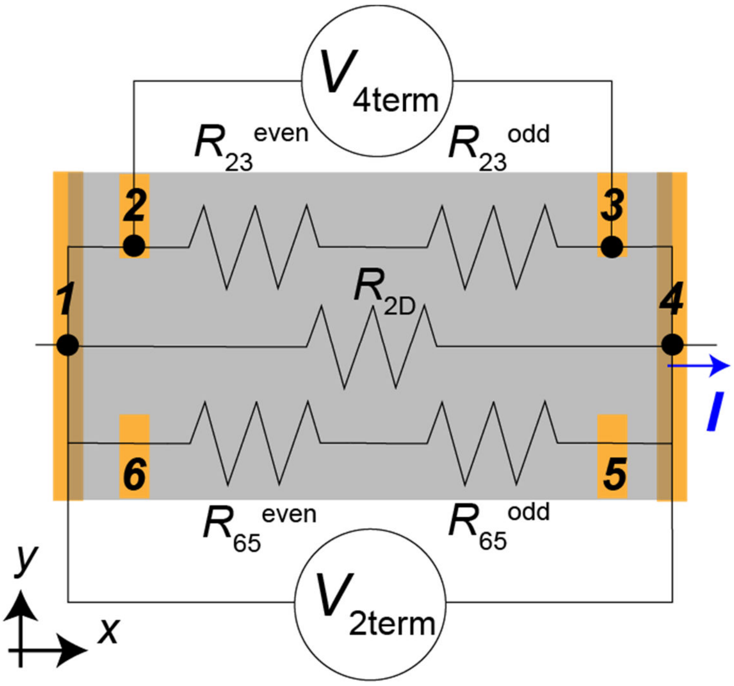

Supplementary Fig. 7| Equivalent circuit model for the OMR in two- and four-terminal measurements. Resistor network diagram representing our InAs/(Ga,Fe)Sb device and schematic diagram of the top view of our Hall bar device. $R_{23(65)}^{\mathrm{even}}$ and $R_{23(65)}^{\mathrm{odd}}$ represent the resistance components that are even and odd functions of the external magnetic field, respectively, observed in the upper (lower) terminals.

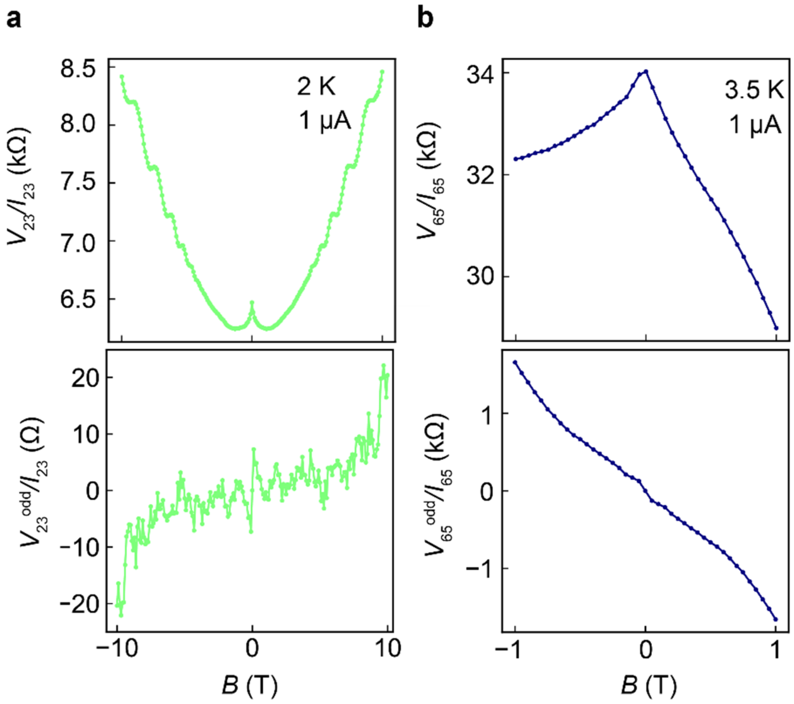

Supplementary Fig. 8| Two-terminal measurement of D1 and D2. a, Two-terminal measurement (V_{23}/I_{23}) of device D1 at 2 K with 1μA. **b**, Two-terminal measurement (V_{65}/I_{65}) of device D2 at 3.5 K with 1μA.

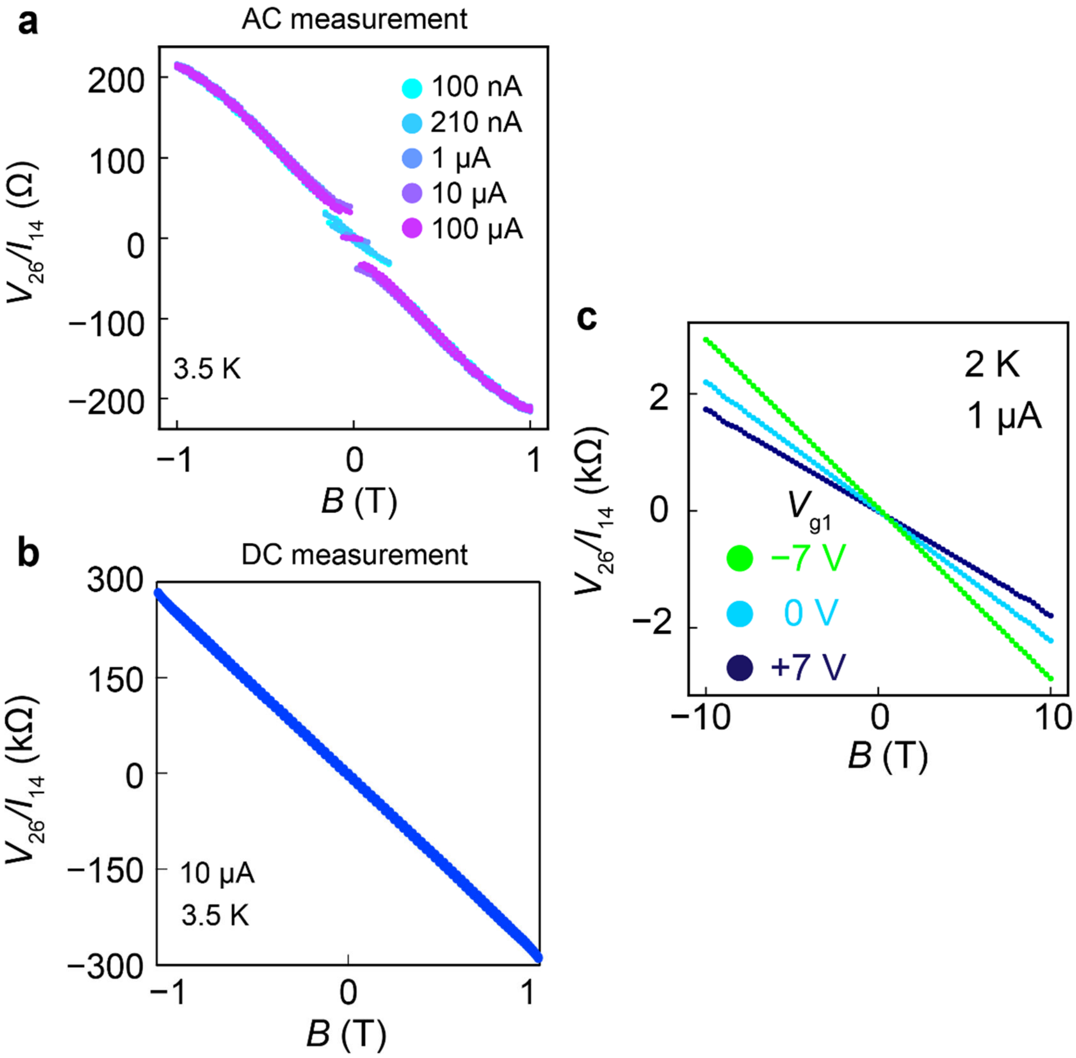

Supplementary Fig. 9| Current dependence of the Hall resistance a, Current dependence of the Hall resistance (V_{26}/I_{14}) of device D1 measured by the lock-in technique with 5261 Hz at 3.5 K. **b**, Hall resistance measured using DC current at 3.5 K. When we measured the Hall resistance with DC current, the jumps near zero field does not appear. We note that the difference of the magnitude of Hall resistance between **a** and **b** is due to the difference of the samples. **c**, Hall resistance (V_{26}/I_{14}) *vs*. perpendicular magnetic field B of device D2 measured at various gate voltage V_{g1} (applied to gate G1) at 2 K with 1 μA. As shown in Fig. 3b, the OMR changes its polarity by switching V_{g1} from +7 V to −7 V. However, the Hall resistance does not exhibit the sign change in this V_{g1} region.

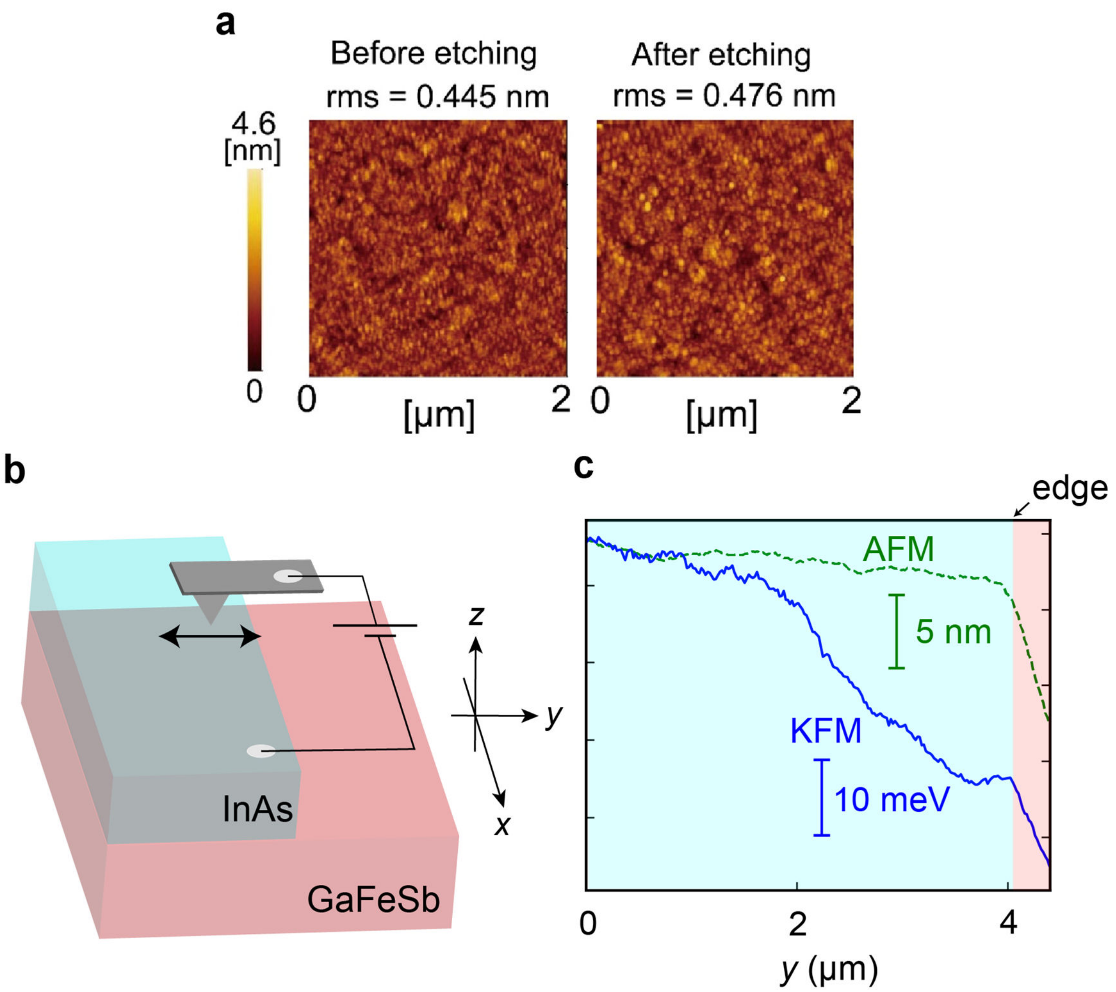

Supplementary Fig. 10| Atomic force microscope (AFM) and Kelvin force microscopy (KFM) measurement of InAs/(Ga,Fe)Sb a, Surface morphology measured by AFM of the InAs/(Ga,Fe)Sb wafer before (left) and after (right) the etching using Ar ion milling. **b**, Schematic image of the configuration of the KFM measurement. **c**, KFM (blue solid line) and AFM (green dashed line) results in the *y* direction sweep. The red shaded area is the place where the tip goes through the edge of the sample.

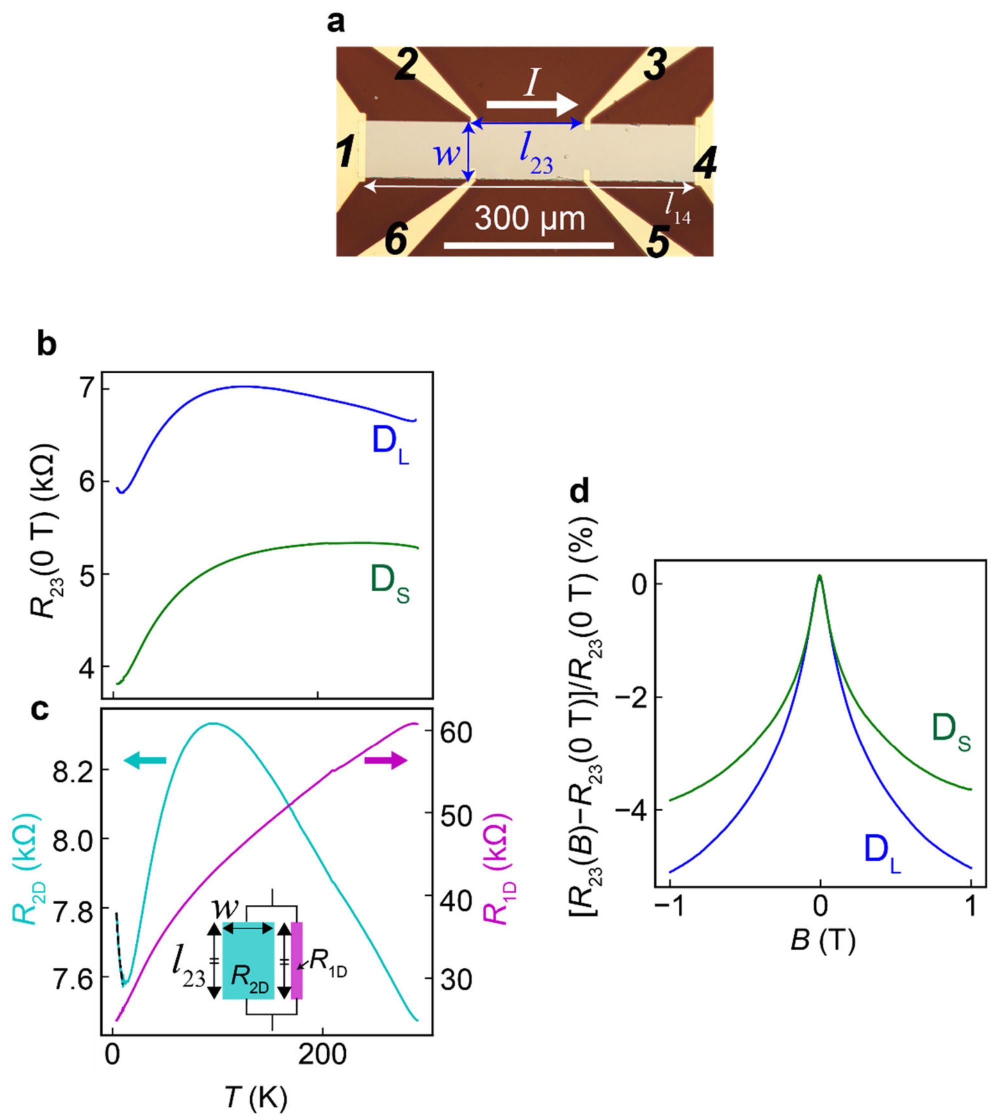

Supplementary Fig. 11| Device-size effect on the 1D and 2D transport. a, Optical microscope image (same as Fig. 1b in the main manuscript) of the Hall bar device D_L. Here, w and l_{23} indicate the width and the distance between “2” and “3” electrodes, respectively. **b**, Temperature (T) dependence of R_{23}(0 T) of device D_L (blue) and device D_S (green). **c**, T dependence of R_{2D} (cyan) and R_{1D} (purple) in D_L. The inset shows the schematic resistor network representing R_{23}. The 2D resistance R_{2D} has the width w and length l_{23}, and the 1D resistance R_{1D} has the same length. The black dashed line at $T <$ ~10 K is the fitting result using a logarithmic function, which is characteristic of the Kondo effect ($R_{2D} = R_{c0} - R_{c1} \ln T$, where R_{c0} and R_{c1} are fitting parameters). **d**, Normalized magnetoresistance by the zero-field resistance, $[R_{23}(B)-R_{23}(0\ \mathrm{T})]\ /\ R_{23}(0\ \mathrm{T})$, of D_L (blue) and D_S (green).

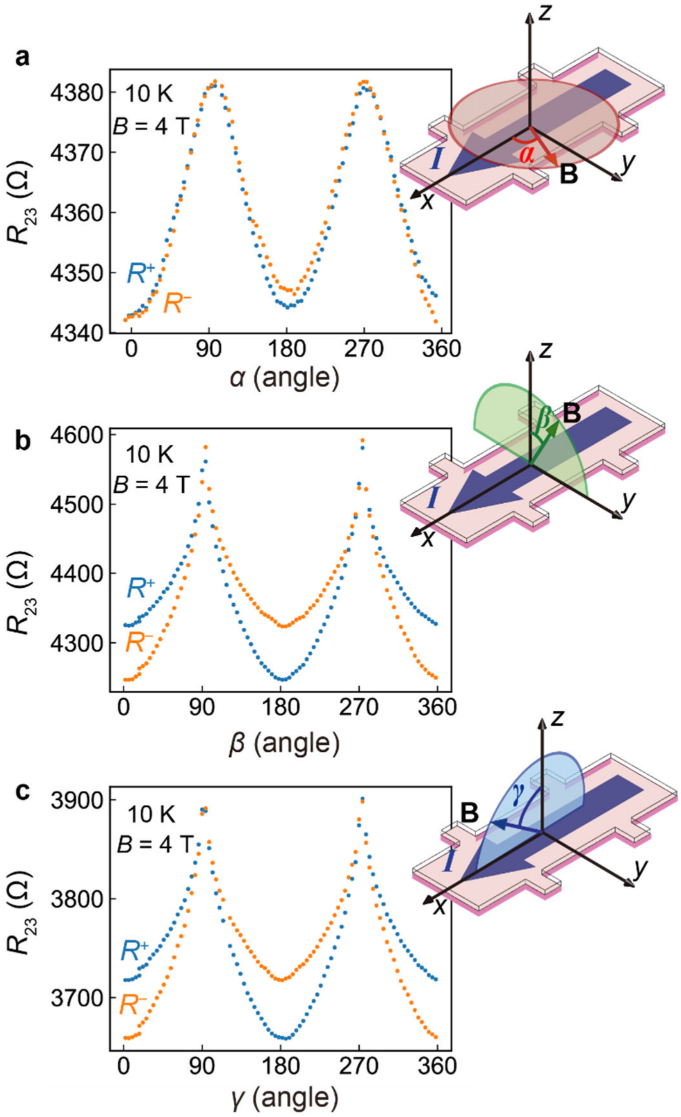

Supplementary Fig. 12| Angle dependence of the OMR in InAs/(Ga,Fe)Sb a, b, and **c** Magnetic-field angle dependence of R_{23} ($=V_{23}/I_{14}$) in *xy*, *yz* and *zx* rotation. As shown in the schematic image, each rotation angle is defined as α, β, and γ in the *xy*, *yz* and *zx* plane, respectively. Blue and orange dots indicate R^+ and R^-, respectively, where R^+ and R^- are defined as R_{23} when the magnetic field B is positive and negative, respectively. The difference between R^+ and R^- corresponds to the OMR magnitude.

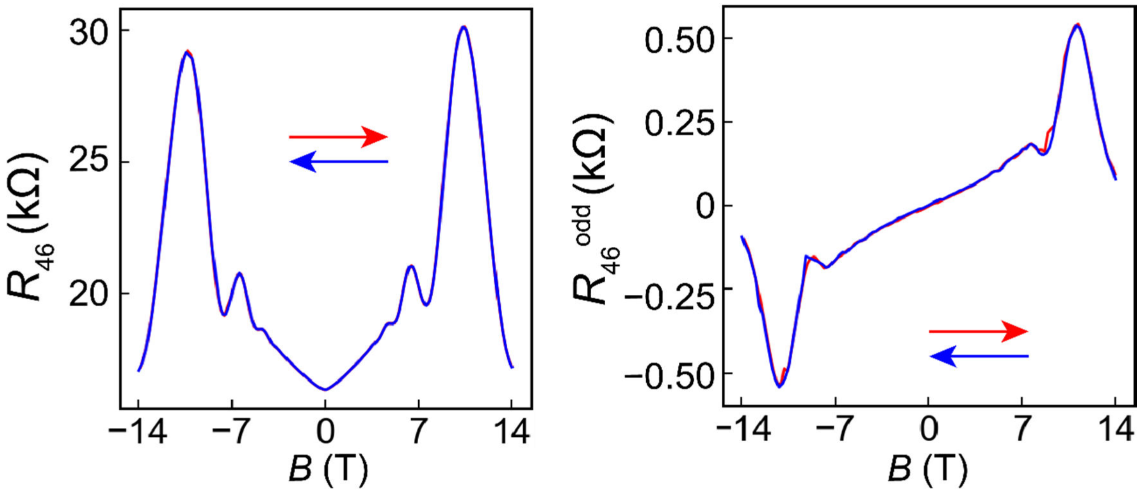

Supplementary Fig. 13| Magnetoresistance of a nonmagnetic InAs/GaSb bilayer (left side graph) and its odd component (right side graph) at 2 K. The red and blue arrows indicate sweep direction of the magnetic field. The OMR magnitude is less than 1.8% at 14 T, much smaller than that (13.5% at 10 T) in the InAs/(Ga,Fe)Sb bilayer.

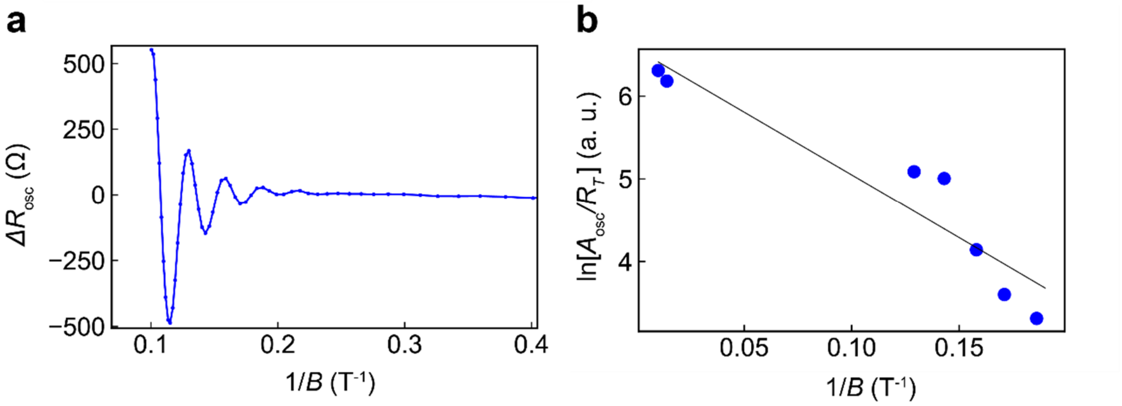

Supplementary Fig. 14| Dingle plot for sample A a, Oscillating component ΔR_{osc} of sample A extracted from the magnetotransport data shown in Fig. 1c in the main manuscript. The background signal is fitted by a third polynomial function and subtracted from the raw data. **b**, Dingle plot from the data in **a**. A_{osc} is the peak value of the SdH oscillation. R_T is the temperature reduction factor: $R_T = \sinh X / X, X = 2\pi k_B T / \hbar\omega_c$. Here, k_B is Boltzmann's constant, and ω_c (= eB/m^*) is the cyclotron angular frequency. The black line indicates the fitting of the Dingle plot.

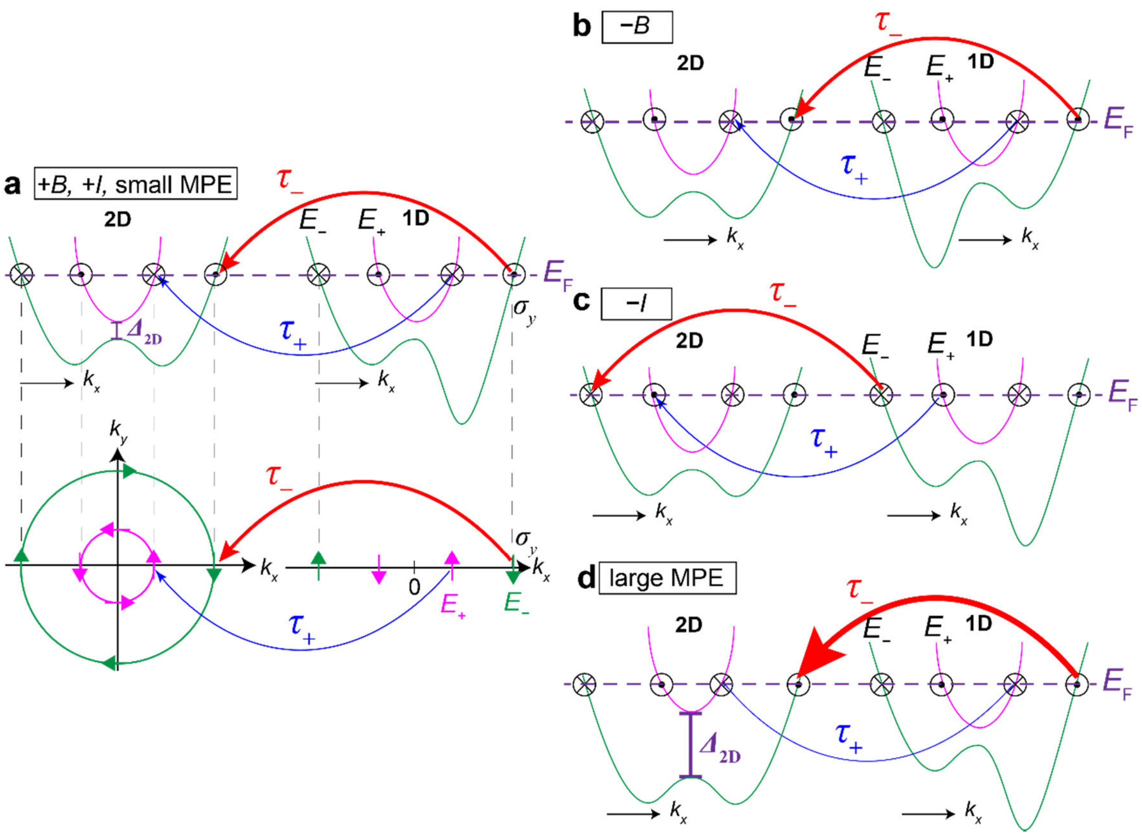

Supplementary Fig. 15| Mechanism of asymmetric scattering. a, Schematic energy band dispersions (upper panel) and their Fermi surfaces (lower panel) in the k_x direction of the 1D edge (right) and 2D (left) channels in the InAs layer, where a magnetic field B is applied in the z direction perpendicular to the plane. The horizontal purple dashed line indicates the Fermi level E_F. Here we consider that the scattering between the 1D and 2D channels is only allowed within the same chirality (σ_y), which are indicated by the blue and red arrows. These scattering processes have different relaxation times of τ_+ and τ_-, respectively, because of the different density of states between E_+ and E_- at E_F. **b**, Energy band dispersions when the magnetic field B is reversed. The chirality of E_+ and E_-, which is determined by the Rashba SOI, is unchanged. Therefore, α (= τ_-/τ_+) remains unchanged. **c**, When we flip the current I, the scattering occurs in the $-k_x$ region. In this case, α also remains unchanged, thus the OMR in the InAs/(Ga,Fe)Sb bilayer does not depend on the current direction. **d**, The MPE opens a gap (= Δ_{2D}) in the 2D channel. This affects the DOS of each chirality in the 2D channel, which enhances the imbalance of τ_+ and τ_- and leads to larger OMR.

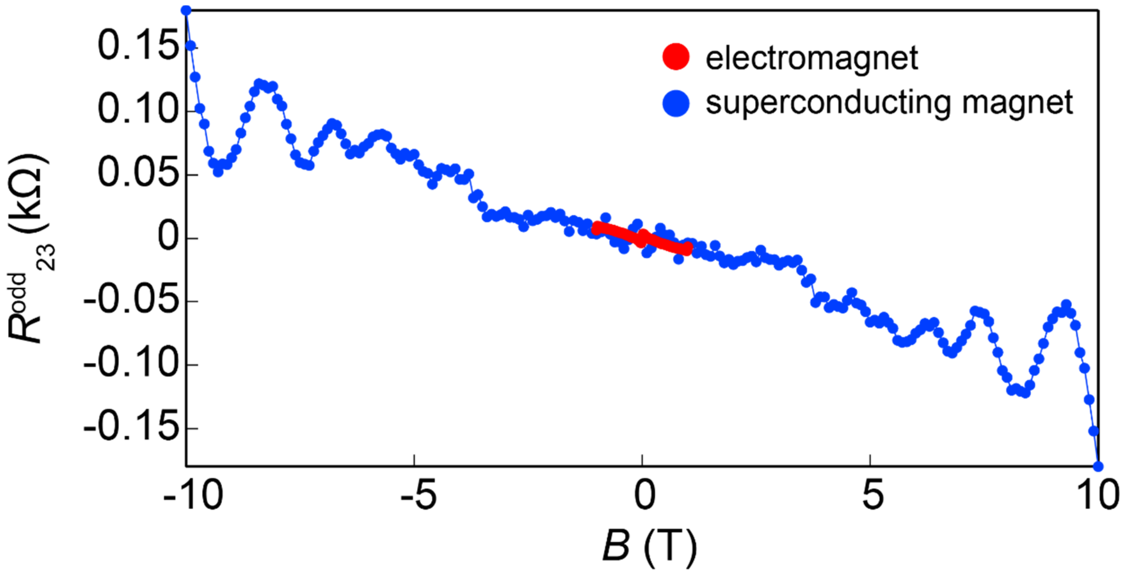

Supplementary Fig. 16| Magnetic sweeping effect on OMR Comparison of odd components of magnetoresistance ($R_{23}^{odd} = [R_{23}(B) - R_{23}(-B)]/2$, B is the magnetic field) measured by two different transport machines with electromagnet (red) and superconducting magnet (blue). These data are measured at 3.5 K and 2 K, respectively with 1 μA. B is applied perpendicular to the plane.

References

S1 Lifshitz, I. M. & Kosevich, A. M., Theory of magnetic susceptibility in metals at low temperature. *Sov. Phys. JETP* **2**, 636 (1956).

S 2 Shoenberg, D., Magnetic oscillations in metals (Cambridge University Press, Cambridge, UK, 1984)

S3 Takiguchi, K., Anh, L. D. *et al.*, Giant gate-controlled proximity magnetoresistance in semiconductor-based ferromagnetic–non-magnetic bilayers. *Nat. Phys.* **15,** 1134 (2019).

S4 Knox, C. S. *et al.*, Deconvolution of Rashba and Dresselhaus spin-orbit coupling by crystal axis dependent measurements of coupled InAs/GaSb quantum wells. *Phys. Rev. B* **98**, 155323 (2018).

S5 Shobhit, G. *et al.*, Temperature dependence of magnetic anisotropy in heavily Fe-doped ferromagnetic semiconductor (Ga,Fe)Sb. *J. Appl. Phys*. **127**, 023904 (2020).

S6 Yuan, Y. *et al.*, Nematicity of correlated systems driven by anisotropic chemical phase separation. *Phys. Rev. Mat.* **2**, 114601 (2018).

S7 C. A. Mead and W. G. Spitzer, Fermi Level Position at Semiconductor Surfaces *Phys. Rev. Lett.* **10**, 472 (1963)

S8 L. F. J. Piper et al., Electron depletion at InAs free surfaces: Doping-induced acceptorlike gap states *Phys. Rev. B* **73**, 195321 (2006)

S9 Cartoixà, X., Ting, D.Z.-Y. & McGill, T. C., Theoretical investigations of spin splittings and optimization of the Rashba coefficient in asymmetric AlSb/InAs/GaSb Heterostructures. *J. Comput. Electron.* **1**, 141 (2002).

S10 Tokura, Y. & Nagaosa, N. Nonreciprocal responses from non-centrosymmetric quantum materials. *Nat. Commun.* **9**, 3740 (2018).

S11 Zhang, S. S. L. & Vignale, G. Theory of unidirectional spin Hall magnetoresistance in heavy-metal/ferromagnetic-metal bilayers. *Phys. Rev. B* **94**, 140411(R) (2016).

S12 Rikken, G. L. J. A., Fölling, J. & Wyder, P. Electrical magnetochiral anisotropy. *Phys. Rev. Lett.* **87**, 236602 (2001).

S13 Rikken, G. L. J. A. & Wyder, P. Magnetoelectric anisotropy in diffusive transport. *Phys. Rev. Lett.* **94**, 016601 (2005).

S14 Singha, R., Satpati, B. & Mandal, P. Fermi surface topology and signature of surface Dirac nodes in LaBi. *Sci. Rep.* **7**, 6321 (2017).

S15 Abrikosov, A. A. Quantum linear magnetoresistance; solution of an old mystery. *J. Phys. A. Math. Gen.* **36**, 9119–9131 (2003).

S16 Xiao, C. *et al.* Linear magnetoresistance induced by intra-scattering semiclassics of Bloch electrons. *Phys. Rev. B* **101**, 201410 (2020).

S17 Zyuzin, V. A. Linear magnetoconductivity in magnetic metals. *Phys. Rev. B* **104**, L140407 (2021).

S18 Moubah, R. *et al.*, Antisymmetric magnetoresistance in SmCo5 amorphous films with imprinted in-plane magnetic anisotropy. *J. Appl. Phys.* **115**, 053911 (2014).

S19 Wang, Y *et al.*, Antisymmetric linear magnetoresistance and the planar Hall effect. *Nat. Commun.* **11,** 216 (2020).

S20 Fujita, T. C. *et al.*, Odd-parity magnetoresistance in pyrochlore iridate thin films with broken time-reversal symmetry. *Sci. Rep.* **5,** 9711 (2019).

S21 Albarakati, S *et al.*, Antisymmetric magnetoresistance in van der Waals Fe_3GeTe_2 /graphite/Fe_3GeTe_2 trilayer heterostructures. *Sci. Adv.* **5,** eaaw0409 (2019).
